# Lean Six Sigma handoff process between operating room and pediatric ICU: improvement in patient safety, efficiency and effectiveness

**DOI:** 10.1186/cc14603

**Published:** 2015-03-16

**Authors:** SJ Gleich, ME Nemergut, AA Stans, DT Haile, SA Feigal, AL Heinrich, CL Bosley, JW Ward, S Tripathi

**Affiliations:** 1 Mayo Clinic, Rochester, MN, USA

## Introduction

This Six Sigma project was initiated to evaluate and improve the transfer of care of patients from the OR to the ICU. Medical errors are responsible for billions of dollars in increased healthcare spending. Miscommunication among healthcare providers is a major contributor to these errors, with handoffs a particularly vulnerable period in the care process. At our institution, surgical patients with scheduled admissions to the ICU are first recovered in the postanesthesia care unit (PACU). With this process, multiple, unstructured, and individual handoffs occur in parallel between providers, which may lead to communication errors, differential information sharing, content variability, care delays, and inefficiency.

## Methods

A multidisciplinary QI project was initiated with input from the ICU, anesthesia and surgical services. A series of PDSA cycles were conducted, which began by defining the current process via direct observation and value stream mapping of orthopedic and neurosurgical patients. A new process was then introduced, including direct transfer of the patient to the ICU and a single, structured, bedside report between all care providers. A standardized handoff tool was implemented. We used process times, wait times and information content as process measures and handoff errors as outcome measures. A 10-point satisfaction score was also measured.

## Results

Following implementation of the new transfer process, the average wait time decreased by 58 minutes, process time decreased by 9 minutes, and lead time decreased by 66.5 minutes. The handoff error rate decreased by 1.3 errors/patient and first-time quality rate increased by 67%. Staff satisfaction improved from 48% to 73%. By elimination of the PACU stay, the costs involved in admission to the PACU were deferred.

## Conclusion

A single, multidisciplinary bedside handoff process between the OR and ICU leads to cost and time savings. By elimination of redundant, nonvalue-added processes, less opportunity for medical errors occurred, with substantial improvements in first-time quality. Such a process can be successfully attained while affecting staff satisfaction positively.

